# Indium Doped Zinc Oxide Thin Films Deposited by Ultrasonic Chemical Spray Technique, Starting from Zinc Acetylacetonate and Indium Chloride

**DOI:** 10.3390/ma7075038

**Published:** 2014-07-04

**Authors:** Rajesh Biswal, Arturo Maldonado, Jaime Vega-Pérez, Dwight Roberto Acosta, María De La Luz Olvera

**Affiliations:** 1 Departamento de Ingeniería Eléctrica, Centro de Investigación y de Estudios Avanzados del Instituto Politécnico Nacional, Sección de Electrónica del Estado Sólido, Código Postal 07360, México D.F., Mexico; E-Mails: amaldo@cinvestav.mx (A.M.); molvera@cinvestav.mx (M.D.L.L.O.); 2 Escuela Superior de Ingeniería Mecánica y Eléctrica, Unidad Ticoman del Instituto Politécnico Nacional, Código Postal 07340, México D.F., Mexico; E-Mail: jvegap@ipn.mx; 3 Instituto de Física, Universidad Nacional Autónoma de México, Código Postal 04510, México D.F., Mexico; E-Mail: dacosta@fisica.unam.mx

**Keywords:** thin films, optical properties, electrical conductivity

## Abstract

The physical characteristics of ultrasonically sprayed indium-doped zinc oxide (ZnO:In) thin films, with electrical resistivity as low as 3.42 × 10^−3^ Ω·cm and high optical transmittance, in the visible range, of 50%–70% is presented. Zinc acetylacetonate and indium chloride were used as the organometallic zinc precursor and the doping source, respectively, achieving ZnO:In thin films with growth rate in the order of 100 nm/min. The effects of both indium concentration and the substrate temperature on the structural, morphological, optical, and electrical characteristics were measured. All the films were polycrystalline, fitting well with hexagonal wurtzite type ZnO. A switching in preferential growth, from (002) to (101) planes for indium doped samples were observed. The surface morphology of the films showed a change from hexagonal slices to triangle shaped grains as the indium concentration increases. Potential applications as transparent conductive electrodes based on the resulting low electrical resistance and high optical transparency of the studied samples are considered.

## 1. Introduction

Zinc oxide (ZnO) is a wide band gap (3.37 eV at room temperature) semiconductor with high exciton binding energy (60 meV) with multiple functionalities. Characteristics such as high transmittance in visible spectral region, adjustable conductivity, high catalytic activity, piezoelectricity and a high chemical stability against reducing plasma [1] make the ZnO a semiconductor notably useful for both in nanostructure and in thin film forms. ZnO thin films have been utilized for several applications such as transparent conductive oxide (TCO) in thin film solar cells, antireflective coatings [2,3] and TFTs [4]. In recent times, significant technical advancement has been reported for utilizing ZnO as TCO, over fluorine doped tin oxide and expensive indium oxide [5]. ZnO thin films of varied characteristics have been reported based on a wide number of physical and chemical techniques [6]. Due to the high cost of vacuum equipment and their corresponding maintenance, needed in physical deposition techniques, there is a growing interest in the development of reliable chemical process for depositing quality thin films. However, depending on the required features, both physical and chemical techniques have been utilized for fabricating ZnO thin films.

In the manufacturing of quality ZnO thin films such as TCO, a substantial effort has been focused on decreasing the electrical resistivity of the films with metal doping, particularly of Group III elements of the periodic table. It is worth mentioning that, successful results have been achieved: ZnO thin films with a low resistivity in the order of (~2 × 10^−4^ Ω·cm) have been reached by physical deposition processes [7]. While this resistivity value, considered almost the theoretical limit in ZnO thin films, may be obtained straightforwardly by sophisticated physical techniques, a similar result based on simple and economical, chemical deposition techniques is still in progress. Hence, thorough studies based on the effect of deposition variables, in chemical techniques, are required in order to achieve high quality films competitive with those obtained by physical techniques. Among the chemical deposition techniques reported for manufacturing good quality ZnO thin films, chemical spray is distinguished for its simple set-up and easy adaptation for large scale production [8]. The lowest resistivity reached for chemically sprayed ZnO:In thin films deposited over sodocalcic glass substrates is around 2 × 10^−3^ Ω·cm [8,9]. While it is hard to obtain low resistivity values in undoped ZnO films deposited by chemical techniques, different efforts have been made to improve the conductivity with intentional doping.

It is worth mentioning that the chemical spray technique has also been improved significantly in recent years, assisted by diverse innovations in the atomization process like ultrasonic excitation, obtaining more uniform, compact thin films having smoother surfaces with enhanced transport properties [10]. Atomization of starting solution through ultrasonic excitation induces fog formation, decreasing transportation rates of precursors as compared to the pneumatic version, causing the deposition process to resemble chemical vapor deposition (CVD). In contrast, the pneumatic atomization process involves instantaneous wetting and evaporation, where flow rates as large as 12 mL/min are directed continuously towards the hot substrate [11]. Also, it has been shown that the nature of a precursor plays a significant role in the final characteristics of ZnO thin films [12] deposited by chemical spray. Differences in surface morphology of ultrasonic sprayed ZnO thin films due to the addition of either organic or inorganic acids in the starting solution, as stabilizers have also been reported [13]. Although the ultrasonic spraying has showed to be a better alternative for the deposition of metal oxide thin films with improved properties, information on the effect of solution composition on their morphology and other physical characteristics is still under progress. It is noteworthy that zinc acetate has usually been employed as a zinc precursor for the deposition of ZnO films by ultrasonic chemical spray [14,15] but there have been only a few reports on the deposition of ZnO thin films based on zinc acetylacetonate [16] as zinc precursor.

Since an ultrasonic spray process resembles a CVD system, an organometallic precursor should be adequate for film deposition under near equilibrium conditions of mass transport and substrate temperature. This might also be an alternative for achieving low resistivity ZnO thin films, matching well with those developed by sophisticated physical techniques.

In this work, we present the deposition of ZnO:In thin films on sodocalcic glass substrates, starting from zinc acetylacetonate and indium chloride as zinc and indium precursors, respectively. The effect of indium concentration and substrate temperature on the structural, morphological, electrical and optical properties of the films has been studied.

## 2. Experimental Details

The ZnO thin films were deposited starting from a 0.2 M solution of zinc acetylacetonate dissolved homogeneously into a non-aqueous mixture of acetic acid and methanol (1.25:48.75 volume proportions, respectively). Indium (III) chloride, was selected as the doping source, and different [In]/[In+Zn] atomic per cent ratios, *i.e.*, 1.5, 3.0 and 4.0 at% were tested. The reason why these indium doping values were selected is due to the fact that the ZnO thin films deposited with doping concentrations out of this range showed higher resistivity values. The deposition system used in this work was based on a home-made commercial humidifier with a frequency of 1.2 MHz adapted for atomization of the starting solution. Sodocalcic glass plates of about 2.5 × 5.0 cm^2^ sizes with a thickness of 1.1 mm, cleaned previously were utilized as substrates. A molten tin bath was used for substrate heating, and the temperature of the bath was electronically controlled and monitored by a K-type thermocouple. Substrate temperatures selected were 400, 425 and 450 °C, within ±1 °C variation.

The selection of the temperature range was based on the fact that depositions at lower substrate temperatures yielded highly resistive films, whereas at temperatures higher than 450 °C, powdery finished surfaces were obtained due to the initiation of gas phase reaction. The solution flux rate was in the order of 1.0 mL/min. Nitrogen was used as carrier gas. Under these conditions, the ZnO:In thin films were deposited for 10 min at a growth rate of 100 nm/min, in order to obtain a film thickness of about 1 μm. The deposited thin films presented an apparent homogeneous surface and very good adhesion with the substrate. Structural analysis of the samples was performed through X-ray diffraction (XRD) measurements in θ–2θ mode using the CuKα (λ = 1.5406 Å) radiation with a Siemens Kristalloflex diffractometer (Siemens, Berlin, Germany). The morphology of the films was studied with a JEOL JSM-6510LV (Jeol, Tokyo, Japan) scanning electron microscope (SEM) operating at 15 KeV. Optical transmittance spectra of the ZnO:In thin films at normal incidence were recorded in a Shimadzu 2401 PC double-beam spectrophotometer (Shimadzu Corporation, Kyoto, Japan) in the 300–1000 nm spectral range, and no correction of glass substrate was made. The sheet resistance, *R*_s_, of the as-grown ZnO:In thin films were measured by the four-point probe technique, taking into account the adequate geometrical corrections. Subsequently, with these resistance values and the film thicknesses, *t*, the electrical resistivities were estimated, ρ = *R*_s_*·t*. The structural and morphological characterization was performed in films deposited at 450 °C from starting solutions with different In content, namely, 0, 1.5, 3, and 4 at%, whereas optical characterization was limited to films with [In]/[In+Zn] = 3 at% but deposited at different substrate temperatures. Finally, electrical characterization was done on all deposited samples.

## 3. Results and Discussion

### 3.1. Structure

**Figure 1:**
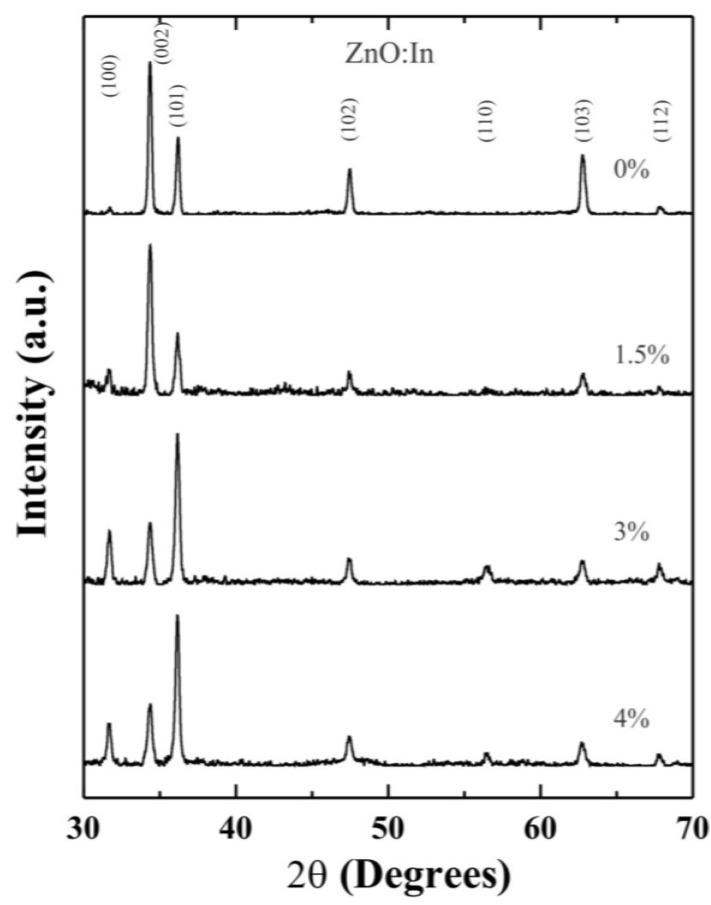
XRD patterns of ZnO:In thin films containing different nominal indium concentrations deposited at *T*_s_ = 450 °C.

Figure 1 shows the X-ray diffraction spectra of undoped and indium doped ZnO thin films prepared from starting solutions with different indium concentrations, at a substrate temperature, *T*_s_ = 450 °C. It can be seen that all spectra exhibit polycrystalline structure that fit well with the hexagonal wurtzite phase of ZnO (JCPDS No. 36-1451).

In the case of undoped and 1.5 at% In-doped ZnO thin films, the intensity of the peak associated with (002) plane prevails over the rest, showing a well-defined preferential growth. However, in the case of the samples doped at 3 and 4 at% the preferential orientation is switched to the (101) plane. In the spectra, only small changes in the intensity of the peaks of undoped and 1.5% were registered. This result points out that low In concentrations in the starting solutions leads to less incorporation of In into the films; therefore, In does not significantly affect the crystalline structure of ZnO. However, the geometry of the grains of the films may have been affected by the change in preferential growth, making it necessary to study the surface morphology of the films in order to evaluate such changes. The crystallite sizes, *D*, were determined by using the Scherer’s equation [17], given by:

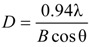
(1)
where λ = 0.15418 nm, is the wavelength of the CuKα incident radiation in nm, *B* is the full width at half-maximum of the particular diffraction line, and θ is the Bragg diffraction angle in radians. The average size varied from 36.2 to 43.5 nm; however the values do not present a clear tendency with the In concentration in the starting solutions.

### 3.2. Morphology

**Figure 2:**
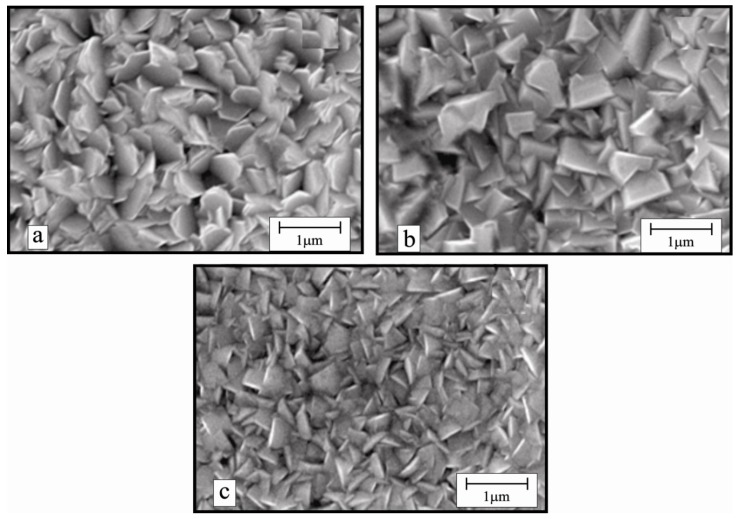
Typical SEM images of ZnO:In thin films prepared at (**a**) 1.5; (**b**) 3.0; and (**c**) 4.0 at% nominal indium concentrations. All the samples were grown at *T*_s_ = 450 °C.

Figure 2 shows the typical SEM micrographs of the ZnO:In thin films prepared at *T*_s_ = 450 °C, with different indium contents.

As it can be seen, the film deposited from a solution containing a ratio of [In]/[In+Zn] = 1.5 at% revealed well-defined morphology with stacked hexagonal slices or plate-like structures with an average diameter of 250 nm. However, further increase in the [In]/[In+Zn] ratio changes the morphology of the films to irregular rectangular grains with embedded pyramidal structures (300–800 nm) as in the case of films deposited from a solution containing a ratio of [In]/[In+Zn] = 3.0 at%. An increase of indium concentration in the starting solution, to 4.0 at% also modifies the surface morphology of the films as triangular shaped laminar structures of about 400 nm height and 10 nm thicknesses were formed at the surface. It has been found that this triangular surface morphology is an efficient light trapping structure which is useful for silicon thin film solar cells [18].

### 3.3. Electrical Characteristics

Electrical conductivity of all the samples were measured by the four-point probe method by considering the geometry of electrical contacts located over them. It was observed that, with the increase in the substrate temperature, the resistivity of the ZnO:In thin films decreases, reaching a minimum value, for a fixed [In]/[In+Zn] ratio in the starting solution. This trend of the resistivity of the films as a function of the substrate temperature has been explained on the basis of stoichiometry deviations. As in the regime of low substrate temperatures, it is considered that the synthesis reaction of ZnO is not complete. Further increase in the substrate temperature leads to an increase in oxygen vacancies and/or zinc interstitial, and hence, the corresponding resistivity decreases. Indium doping enhances conductivity, as it is considered that a fraction of Zn^2+^ ions are replaced by In^3+^ ions, releasing one electron into the lattice, which in turn increases carrier concentration. In the high substrate temperature regime (>450 °C), two factors tend to degrade conductivity, namely, exo-diffusion of alkaline ions coming from the substrate compensate for n-type conduction and undesirable gas phase reaction that takes place, degrading the smooth and uniform surface finish. Regarding the doping effect, there is an optimum value, around [In]/[In+Zn] = 3 at%, related to the solubility limit of In into the ZnO lattice, which is associated with the optimal incorporation of In ions into Zn sites, increasing donor concentration and contributing to a decrease in the resistivity.

From the measured electrical conductivity of the samples presented in Table 1, it is clear to see that a substrate temperature of 450 °C and a ratio of [In]/[In+Zn] = 3.0 at% in the starting solution are optimum parameters to obtain ZnO:In thin films of minimum resistivity. Thin films deposited at those optimum conditions manifest a resistivity as low as 3.42 × 10^−3^ Ω·cm, which is almost one order higher than the lowest resistivity achieved in ZnO thin films so far [7]. Low resistivity of the ZnO:In films containing 3.0 at% nominal indium contents fabricated at *T*_s_ = 450 °C might be associated with their compact surface morphology and bigger crystallite sizes as seen in the SEM images.

**Table 1:** Room temperature electrical resistivity of ZnO:In thin films deposited at different growth conditions.

[In]/[In+Zn]	Electrical Resistivity (Ω·cm)		
	400 °C	425 °C	450 °C
1.5	1.2 × 10^−2^	4.5 × 10^−3^	4.2 × 10^−3^
3	8.4 × 10^−3^	3.6 × 10^−3^	3.42 × 10^−3^
4	2.9 × 10^−2^	1.8 × 10 ^−2^	5.6 × 10^−3^

### 3.4. Optical Characteristics

**Figure 3:**
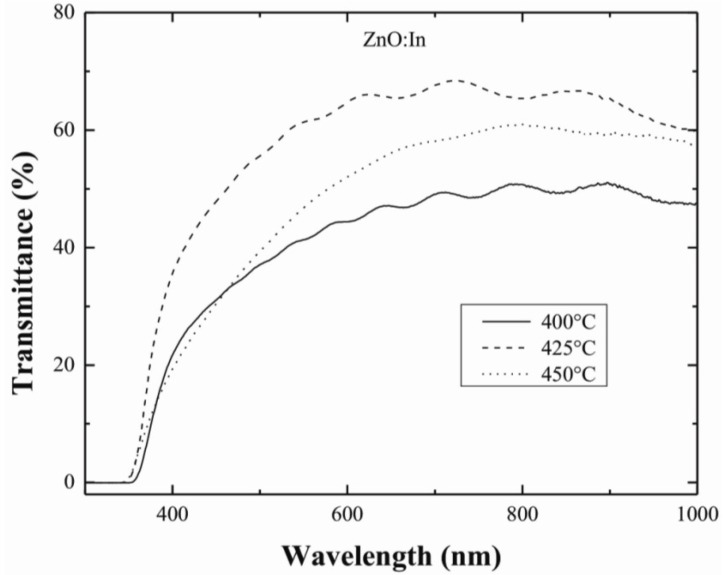
Optical transmittance spectra of ZnO:In thin films deposited at different substrate temperatures for a fixed [In]/[In+Zn] ratio of 3.0 at%.

The optical properties of the ZnO:In films were determined by measuring the optical transmission in the 300–1000 nm wavelength interval. Figure 3 shows the optical transmittance spectra of ZnO:In samples deposited at different substrate temperatures, 400, 425, and 450 °C, for a constant [In]/[In+Zn] ratio of 3.0 at%. It was observed that all the samples revealed a sharp decrease in transmittance in the ultra-violet region (around 375 nm) with a low to average transmittance in the visible region (400–850 nm). Considering direct optical transitions, band gap energy (*E*_g_) of the samples was calculated from a linear extrapolation of the plot (*αhv*)^2^ *versus* *hv*, where *α* is the optical absorption coefficient and *hv* (*h*, Plank’s constant, and *v* is the radiation frequency at the corresponding wavelength) is the energy of the incident photons. The band gap energy values changed from 3.25 to 3.35 eV with the variation of nominal indium contents in the precursor solution. This increase in the band gap of the material can be attributed to the Burstein-Moss effect [19] which states that the increase in the Fermi level in the conduction band leads to the band gap energy broadening as a result of increase in carrier concentration.

## 4. Conclusions

Indium doped zinc oxide thin films thin films were successfully deposited over cheap glass substrates by the ultrasonic spray technique. The effects of substrate temperature and dopant concentration on the physical properties of Indium-doped zinc oxide thin films were investigated. Starting from zinc acetylacetonate as a zinc precursor, ZnO:In thin films, deposited at *T*_s_ = 450 °C with nominal [In]/[In+Zn] = 3.0 at% indium concentration in the starting solution revealed electrical resistivity as low as 3.42 × 10^−3^ Ω·cm. The obtained films present a hexagonal wurtzite structure where the crystallites are preferentially oriented along the (002) planes, which changes according to the doping concentration. While the substrate temperature plays a significant role in controlling the crystallite size of ZnO:In films, concentration of indium ions in starting solution affects their crystallite orientation and room temperature electrical resistivity. SEM images of films deposited at 450 °C using different In concentrations show clear and uniform grains. Low resistivity and a moderate optical transmittance of our ZnO:In thin films grown at optimum growth conditions make them potential candidates for application as TCOs in thin film solar cells, though a considerable amount of work has to be done to control the surface morphology of the films in order to have a better control on the transport properties.
